# Pharmacogenetics of Anti-Diabetes Drugs

**DOI:** 10.3390/ph3082610

**Published:** 2010-08-13

**Authors:** Johanna K. DiStefano, Richard M. Watanabe

**Affiliations:** 1 Metabolic Diseases Division, Translational Genomics Research Institute, 445 N. 5th Street, Phoenix, AZ 85004, USA; 2 Departments of Preventive Medicine and Physiology & Biophysics, Keck School of Medicine, University of Southern California, Los Angeles, CA, USA; E-Mail: rwatanab@usc.edu (R.M.W.)

**Keywords:** pharmacogenetics, biguanides, sufonylureas, thiazolidinediones, type 2 diabetes mellitus, drug response, association analysis, candidate gene

## Abstract

A variety of treatment modalities exist for individuals with type 2 diabetes mellitus (T2D). In addition to dietary and physical activity interventions, T2D is also treated pharmacologically with nine major classes of approved drugs. These medications include insulin and its analogues, sulfonylureas, biguanides, thiazolidinediones (TZDs), meglitinides, α-glucosidase inhibitors, amylin analogues, incretin hormone mimetics, and dipeptidyl peptidase 4 (DPP4) inhibitors. Pharmacological treatment strategies for T2D are typically based on efficacy, yet favorable responses to such therapeutics are oftentimes variable and difficult to predict. Characterization of drug response is expected to substantially enhance our ability to provide patients with the most effective treatment strategy given their individual backgrounds, yet pharmacogenetic study of diabetes medications is still in its infancy. To date, major pharmacogenetic studies have focused on response to sulfonylureas, biguanides, and TZDs. Here, we provide a comprehensive review of pharmacogenetics investigations of these specific anti-diabetes medications. We focus not only on the results of these studies, but also on how experimental design, study sample issues, and definition of ‘response’ can significantly impact our interpretation of findings. Understanding the pharmacogenetics of anti-diabetes medications will provide critical baseline information for the development and implementation of genetic screening into therapeutic decision making, and lay the foundation for “individualized medicine” for patients with T2D.

## 1. Introduction

### 1.1. Pharmacogenetics

There is little doubt that the majority of pharmacologic therapies for common diseases have significantly minimized disease burden and improved the quality of life for affected individuals. In fact, for some diseases, such as type 2 diabetes mellitus (T2D), pharmacologic treatment of at-risk individuals even before manifestation of disease symptoms can significantly reduce disease risk [1,2,3,4,5]. The efficacy of any pharmacologic therapy is due to a balance between drug action (pharmacodynamics) and clearance (pharmacokinetics), coupled with a minimal adverse effect profile. However, many times the specific biologic mechanism of action for a given drug is unknown, resulting in a relative focus on pharmacokinetics, given the lack of pharmacodynamic knowledge. Furthermore, the very nature of drug development and marketing results in identifying compounds that can be used to cast a wide net to treat a large segment of the diseased population. The unfortunate reality is that very rarely is a given pharmacologic agent 100% efficacious in 100% of treated patients. This has resulted in “tweaking” of compounds or creation of related compounds to improve their applicability to a wider spectrum of patients. 

Pharmacogenetic research, which stems back to the late 1800s (*cf.* [6]), attempts to understand the link between genetic variation and response to drugs. In its infancy, the field was mainly restricted to observations of familial clustering of drug reactions, but the combination of the Human Genome [7,8] and HapMap [9,10] projects has transformed the field to include both the area of pharmacogenomics and a wider spectrum of genetic characteristics beyond single nucleotide polymorphisms (SNPs) in the genome. New genetic variants associated with a variety of common diseases identified using genome-wide association studies (*cf.* National Institutes of Health GWAS Catalog; http://ww.genome.gov/gwastudies/) has elucidated new biological mechanisms underlying not just predisposition to disease, but also response to pharmacologic intervention for disease. Furthermore, genome-wide association studies specifically focused on drug response are now appearing in the literature [11,12,13,14,15]. Coupled with other advances in biomedical research, pharmacogenetics has moved beyond the relative focus on pharmacokinetics to pharmacodynamics. These events bring even closer the prospect of identifying genetic variation that may provide information illuminating which drug at which dose may be most effective for a given individual. This raises the probability of bringing “personalized medicine” to fruition to reduce disease morbidity and mortality, and improve quality of life for individuals with T2D.

### 1.2. Type 2 diabetes mellitus

T2D is a multifactorial, heterogeneous group of disorders characterized by a deficiency or failure in maintaining normal glucose homeostasis [16]. For the most part, T2D results from defects in insulin secretion and insulin action. T2D accounts for the majority of all diagnosed cases of diabetes in adults, and is typically associated with obesity, sedentary lifestyle, older age, family history of diabetes, and ethnicity. Susceptibility to T2D is also modulated by genetic factors, as evidenced by twin studies [17,18,19], familial aggregation [20,21,22], and increased disease risk in ethnic minority populations [21,22,23,24,25].

The prevalence of T2D has increased sharply in recent decades and has tracked with similar increases in the prevalence of obesity, one of the primary risk factors for T2D. Current estimates indicate that diabetes affects 23.6 million people in the United States alone, representing 7.8% of the population, and close to 250 million people worldwide [26]. An additional 57 million individuals living in the United States have a pre-diabetic condition in which impaired glucose tolerance or impaired fasting glucose levels places them at high risk for development of T2D [26]. The prevalence of T2D is also increasing in youth [26]. Historically, type 1, or insulin-dependent, diabetes accounted almost exclusively for all cases of childhood diabetes, but at present, 8-45% of newly diagnosed pediatric patients have T2D [27]. 

Diabetes is the seventh leading cause of death in the United States [26]. T2D is also a risk factor for microvascular complications leading to limb amputations, renal failure, and blindness, as well as other disorders such as hypertension, cardiovascular disease, dyslipidemia, and infections. As such, diabetes significantly contributes to morbidity and mortality in the United States. The treatment of T2D also exerts a huge impact on the health care system. The costs of diabetes in medical expenditures and lost productivity in the United States exceeds $174 billion USD; further, the average medical expenditures among individuals with diagnosed T2D are 2.3 times higher than in those without the disease [26]. Clearly, T2D significantly affects both individual quality of life and public health.

Current therapies in the management of diabetes include lifestyle intervention through diet modification and exercise, as well as oral and injected hypoglycemic agents. Ultimately, the goal of all treatment strategies for T2D is to lower blood glucose concentrations to levels that approximate those representing normal range. Maintenance of near-normal glycemic levels has been shown to lessen the risk for development and progression of disease complications [28]. Pharmacologically, T2D is treated with nine major classes of approved drugs, including insulin and its analogues, sulfonylureas, biguanides, thiazolidinediones (TZDs), meglitinides, α-glucosidase inhibitors, amylin analogues, incretin hormone mimetics, and dipeptidyl peptidase 4 (DPP4) inhibitors.

For many patients with T2D, treatment with anti-hyperglycemic drugs is initially successful, yet over time, monotherapy fails and either addition of a second anti-diabetic agent or transition to insulin becomes necessary to restore acceptable glycemic control. Although glycemic control has improved overall over the past decade, approximately 40% of individuals being treated for T2D do not reach the desired glycosylated hemoglobin (HbA1c) target of < 7% [29] and there is no single agent that yields optimal glucose-lowering effects in all treated patients [30]. In a study of long-term glycemic control in T2D, Kahn *et al.* [31] found a cumulative incidence of monotherapy failure at 5 years of 15% with rosiglitazone (a TZD), 21% with metformin (a biguanide), and 34% with glyburide (a sulfonylurea). In the face of such data with respect to monotherapy, combination therapy is now being implemented to treat T2D. The general strategy of combination therapy is to simultaneously treat multiple components of T2D pathogenesis in a multi-pronged attack to control blood glucose levels. Such therapies include combining multiple monotherapies or using combination drugs such as Metaglip (glipizide and metformin: Merck Santé S.A.S.), Actoplus (pioglitazone and metformin: Takeda Pharmaceuticals) or Janumet (sitagliptin and metformin: Merck & Co). As discussed more fully in the following sections, glycemic response to oral anti-diabetic agents is highly variable; there are a number of factors which contribute to inter-individual differences in drug response including age, sex, disease, drug and food interactions, co-morbidity, and genetic factors [32]. Pharmacogenetics research, which assesses the role of genetic determinants of drug response, promises to yield information that may be used to personalize treatment strategies to insure optimal glucose control in all patients, improve treatment efficacy, and reduce the risk of adverse drug events in susceptible individuals. Here, we aim to provide a comprehensive review of pharmacogenetics investigations of three major classes of anti-diabetes medications: sulfonylureas, biguanides, and TZDs. 

## 2. Pharmacogenetic Studies of Anti-Diabetes Drugs

### 2.1. Sulfonylureas

#### 2.1.1. Background

Sulfonylureas are one of the most widely used classes of oral hypoglycemic agents. The most common sulfonylurea agents are tolbutamide, gliclazide, glibenclamide, and glimepiride, and while most individuals respond well to these drugs, pharmacodynamic response efficacy is variable. For example, 10-20% of treated individuals do not achieve adequate glycemic control using even the highest recommended dose (“*primary sulfonylurea failure*”) and 5-10% of patients with T2D who initially respond to sulfonylurea treatment will subsequently lose the ability to maintain near-normal glycemic levels (“*secondary sulfonylurea failure*”) [33,34]. Further, drug dosages typically need to be increased over time as impairment of insulin secretion occurs, until a second hypoglycemic agent is added or, if all hypoglycemic drugs fail, adding or switching to insulin is indicated. Although failure to respond, or deterioration of, response to sulfonylurea therapy is known to result from a variety of factors including poor dietary and/or physical activity compliance, weight gain, reduction of insulin sensitivity, age of onset, or presence of anti-islet cell and glutamic acid decarboxylase antibodies, the strongest predictor of failure is deterioration of β-cell function [35,36].

Maturity-onset diabetes of the young (MODY) is a rare, autosomal dominant form of diabetes. There are six primary forms of MODY, each a consequence of mutations in six different genes [37]. In addition to the autosomal dominant inheritance, MODY is characterized by onset before the age of 25 and β-cell dysfunction typically in the absence of insulin resistance or obesity. MODY3 arises from mutations in the hepatocyte nuclear factor 1 homeobox A gene (*HNF1A*), and patients with this disease are hyper-sensitive to the hypoglycemic effects of sulfonylureas [38]. In an early case study, Pearson *et al.* [39] identified three MODY3 patients with *HNF1A* mutations, in whom cessation and reintroduction of sulfonylureas caused dramatic changes in HbA1c levels, or severe hypoglycemia, in response to introduction of sulfonylureas into the treatment regimen. A subsequent study found that MODY3 patients had a 5.2-fold or 3.9-fold greater response to gliclazide compared to metformin or patients with T2D, respectively [40]. These patients also had a stronger insulin secretory response to tolbutamide and were more insulin-sensitive compared to individuals with common T2D [40]. 

In a similar series of studies, Pearson *et al.* [41] identified rare heterozygous mutations in the potassium inwardly-rectifying channel, sub-family J, member 11 (*KCNJ11*), more commonly known as the ATP-dependent K^+^ channel, that accounted for 30-58% of cases with permanent diabetes diagnosed in patients < 6 months of age or in neonatal diabetes. These mutations resulted in continual activation of the ATP-dependent K^+^ channel, which prevented insulin secretion by pancreatic β-cells and typically produced a misdiagnosis of type 1 diabetes. This misdiagnosis resulted in patients being improperly treated using conventional insulin therapy. Pearson and colleagues demonstrated that patients with these mutations in *KCNJ11* could be successfully treated with sulfonylureas, rather than insulin. Additional studies identified mutations in the ATP-binding cassette, sub-family C (CFTR/MRP), member 8 gene (*ABCC8*), commonly known as the sulfonylurea receptor, which also results in forms of neonatal diabetes [42]. However, only some patients could be successfully treated with sulfonylureas, with carriers of the F132V mutation having to be maintained on insulin therapy.

Together, the findings from these studies were among the first to demonstrate that the genetic etiology of hyperglycemia may modulate response to hypoglycemia agents. Such results yielded strong implications for patient management and paved the way toward elucidating additional genetic factors that might influence drug response in the treatment of T2D.

#### 2.1.2. Mechanism of action

Sulfonylureas stimulate insulin release from pancreatic β-cells by first binding to the high affinity plasma membrane receptor (SUR1) coupled to an ATP-dependent K+ channel (K_ATP_). This interaction closes the K+ channel, which inhibits potassium efflux and depolarizes the plasma membrane, leading to an opening of voltage-gated calcium channels. Calcium influx, and a corresponding increase in intracellular calcium levels, causes release of insulin from the β-cells. 

##### 2.1.2.1. *KCNJ11* and *ABCC8*

The K_ATP_ channels through which sulfonylureas exert their actions are hetero-octameric protein complexes composed of four high-affinity sulfonylurea receptor (SUR1) subunits coupled to four inwardly-rectifying Kir6.2 subunits. The genes encoding these proteins in humans are the ATP-binding cassette transporter sub-family C member 8 (*ABCC8*) and potassium inwardly-rectifying channel, subfamily J, member 11 (*KCNJ11*) genes, respectively. A number of studies have investigated the role of these genes in relation to hypoglycemia, diabetes, and sulfonylurea failure.

Rare monogenic mutations in *ABCC8* cause neonatal diabetes [43] and may increase susceptibility to T2D [44,45,46,47]. Although *ABCC8* encodes the SUR1 receptor, and as such, represents a logical biological candidate for sulfonylurea response, only a few studies have investigated this gene in relation to drug treatment failure. In one study, 115 Chinese patients with T2D were treated with gliclazide and genotyped for marker rs757110, which is located in exon 33 and causes a serine to alanine substitution at position 1369 [48]. In this study, G allele carriers were more sensitive to gliclazide and experienced greater reductions in HbA1c compared with individuals carrying the TT genotype (1.60% ± 1.39 *vs.* 0.76% ± 1.70, respectively; P = 0.044). This marker was also examined in two independent cohorts of Chinese patients with T2D receiving an 8-week treatment with gliclazide, in whom, individuals carrying the G allele had greater decreases in glucose levels compared with individuals carrying the wild type genotype [49]. The authors also found a trend toward greater HbA1c reduction in patients with the GG genotype compared with homozygous carriers of the wild type genotype, although this association did not quite reach statistical significance (P = 0.06) [49]. In these individuals, mean gliclazide dosage requirements were ~78% in individuals carrying the G allele compared to ~84% in TT homozygous patients [49]. Taken together, these findings provide a rationale for investigating this variant in additional populations and using other sulfonylurea agents.

The *KCNJ11* gene has also been extensively investigated. In animal models, Kir6.2-deficient mice show impaired glucose- and tolbutamide-induced insulin secretion [50], while in humans, *KCNJ11* mutations underlie familial persistent hyperinsulinemic hypoglycemia of infancy [51,52,53] and permanent neonatal diabetes [54], and are associated with common forms of T2D [44,55,56,57,58,59,60,61,62,63,64,65]. 

Of the known *KCNJ11* variants, the most widely studied one is marker rs5219 (C/T), which encodes a glutamate to lysine substitution at position 23 (E23K); the variant K allele is associated with increased risk of T2D. Initial investigations of this variant did not observe evidence for association with sulfonylurea failure in 364 newly diagnosed patients with T2D from the (United Kingdom Prospective Diabetes Study (UKPDS) [57], but a subsequent study [66] in 525 Caucasian individuals with T2D found a higher frequency of the K allele in patients who failed sulfonylurea therapy compared to those who did not (66.8% *vs.* 58.0%, respectively), and glibenclamide-stimulated insulin secretion also tended to be lower in islets from patients carrying the variant K allele, compared to individuals with the homozygous E/E genotype, although this difference was not statistically significant. Differences between genotypes became statistically significant, however, when islets were pre-exposed to high glucose, suggesting that impairment of insulin secretion in response to sulfonylureas in the presence of the E allele is exacerbated by a hyperglycemic milieu. In a third study examining severe sulfonylurea-induced hypoglycemia, Holstein *et al.* [67] found the K allele to be associated with higher HbA1c levels compared with the E allele (P = 0.04), which is consistent with previous findings [66]. 

There are several possible factors that may explain discrepancies between the first and second studies. First, the definitions of secondary failure were different: in the UKPDS, failure was defined as patients who needed additional therapy, regardless of type, to control hyperglycemia, while the second study defined failure solely in terms of progression to insulin therapy. Second, mean duration of therapy with oral agents before failure differed between the two studies (1 yr after randomization in UKPDS *vs.* 12 yr in the second study); the shorter duration of therapy in the UKPDS do not allow for the possibility that some individuals carrying the K allele may be destined to experience secondary failure, but hadn’t yet done so. Third, the class and type of sulfonylurea agent differed between studies (chlorpropamide *vs.* glibenclamide), which, as discussed in the following paragraph, may have implications for response based upon genotype at this marker. Finally, the clinical characteristics of patients differed between studies; the UKPDS recruited newly diagnosed patients who were naïve to oral hypoglycemic agents, while the second study recruited patients with known diabetes. Patients with a new diagnosis would be expected to have better β-cell function compared to patients with a longer duration of T2D, which again, could confound the basis for secondary failure independent of genotype at this locus. Given the strong biological support for this marker in modulating response to sulfonyurea therapy, investigations in large, well-designed study samples are warranted.

Despite the discrepancies among the genetic analyses undertaken to date, *in vitro* studies of the E23K variant of *KCNJ11* and the S1369A variant in *ABCC8* showed that ATP sensitivity of the KATP was lower in the K23/A1369 allele combination compared to that of E23/S1369 [68]. The K23/A1369 variant also displayed 3.5-fold inhibition by gliclazide, but not glibenclamide, suggesting that this variant combination may affect clinical efficacy for some, but not all, sulfonylurea agents. These results are consistent with variable response to different sulfonylurea agents and provide a preliminary rationale for personalizing treatment strategies for individuals who carry the risk genotypes at these loci.

##### 2.1.2.2. *CYP2C9* and *CYP2C19*

Sulfonylureas are metabolized primarily by the cytochrome P450 2C9 enzyme (*CYP2C9*) [69]. Many *CYP2C9* have been identified, but the most common allele is designated as *1, which is the most frequent across populations and is generally considered the wild type allele of the gene [70]. The most studied allelic variants of this gene are Arg144Cys (*i.e.* rs1799853 or CYP2C9*2) and Ile359Leu (*i.e.* rs1057910 or CYP2C9*3), which have respective frequencies of ~11% (*2) and 7% (*3) in Caucasians [70]. Most studies have found that individuals carrying at least one *2 or *3 allele exhibit reduced CYP2C9 activity, while those with either the *2/*3 or *3/*3 genotype show reduced drug-metabolizing activities , with a lower dose requirement, compared with individuals having the wild-type Arg144/Ile359 (CYP2C9*1) allele [71,72,73,74]. Even in healthy volunteers receiving glimepiride, *CYP2C9* genotype altered the pharmacokinetic profile of the drug significantly, with a much slower elimination of glimepiride in individuals carrying the *3 allele compared to those with the *1/*1 genotype [75].

In patients with T2D who received glimepiride, Suzuki *et al*. [76] found larger reductions in HbA1c and higher plasma concentration-time curves for glimepiride in individuals with the *1/*3 genotype compared to those with the *1/*1 genotype. In these individuals, the total clearance of glimepiride was reduced and the mean residence time was longer in patients with the *1/*3 genotype compared with wild type carriers [76]. For patients with T2D receiving tolbutamide, the prescribed dose was lower in individuals carrying the *3 allele compared to those with the wild type genotype [77]. Yet in contrast to the study by Suzuki *et al.* [76], no difference in response to sulfonylurea agents other than tolbutamide (*i.e.* glibenclamide, gliclazide, and glimepiride), was observed between *3 allele carriers and individuals carrying the *1 or *2 alleles, but the number of patients receiving these other drugs was quite small. The mean decrease in glucose levels was 0.3 mmol/L larger for patients with the *1/*2 or *2/*2 genotype and 1.2 mmol/L for the *3 carriers compared to individuals with the wild type genotype, but these differences were not statistically significant. In comparison, a study by Shon *et al.* [78] found lower glucose levels in *1/*3 individuals using tolbutamide, while the findings reported by Kirchheiner *et al.* [79] found that the clearance of glibenclamide in homozygous *3 allele carriers was only 20% that of wild type carriers. This group also found that total 12-hour insulin secretion after a single glibenclamide dose was higher in healthy individuals with the *3 allele compared to those with the *1/*1 genotype. In Chinese individuals with T2D, the average glibenclamide area under the curve (AUC) in individuals with the *1/*3 genotype was twice the amount observed in patients with the *1/*1 genotype. Zhang *et al.* [80] reported similar findings among glibenclamide users. In general, most of the studies of the *CYP2C9* variants and sulfonylurea response utilized quite small study samples, which limits the conclusions that can be drawn from them, but overall, across different sulfonylurea agents and diverse populations, carriers of the variant alleles exhibit decreased sulfonylurea clearance, suggesting that these polymorphisms may be exerting an effect on drug response or metabolism. Additional, larger studies are necessary to unequivocally evaluate the role of these markers in mediating variability in response to sulfonylurea treatment.

CYP2C19, another enzyme with drug metabolizing activity, may also play a role in sulfonylurea metabolism. Two common markers in this gene, *2 (rs4244285) and *3 (rs4986893), produce a non-functional enzyme, and individuals with either allele are referred to as “poor metabolizers” [81]. The *3 allele is more frequent in individuals of Asian ethnicity and not surprisingly, the “poor metabolizer” phenotype is more common in Asians compared to Caucasians, 2-6% *vs.* 10-25%, respectively [82,83]. In healthy Chinese males, the AUC of gliclazide was increased 3.4-fold in “poor metabolizers” compared with carriers of the wild type genotype [80]. The half-life of gliclazide was also prolonged from 15.1 to 44.5 h in “poor metabolizers” [80]. 

##### 2.1.2.3. Other genes

Markers in a few additional genes have also been investigated as modulators of sulfonylurea response, based upon biological candidacy and/or previous findings of association with T2D. Results from investigations of three genes encoding the insulin receptor substrate-1 (*IRS1*), the transcription factor 7-like 2, T-cell specific, HMG-box (*TCF7L2*), and nitric oxide synthase 1 adaptor protein (*NOS1AP*) are briefly presented below.

Early investigations in pancreatic β-cell lines [84] and human pancreatic islets of Langerhans [85] found that genotype at the Gly972Arg marker of *IRS1* affected the level of insulin secretion in response to sulfonylureas. The clinical impact of this marker was also investigated in 477 Caucasians with T2D who were treated with sulfonylurea agents. In these individuals, the genotype frequency of the variant allele (Arg972) was almost twice as high in patients who experienced secondary sulfonylurea failure compared to individuals with controlled glycemia [86]. To our knowledge, this is the sole investigation of this locus in regard to sulfonylurea response. Additional studies will be necessary to determine the extent to which this marker may clinically impact failure to sulfonylurea therapy.

The most replicated locus for susceptibility to T2D is *TCF7L2*, in which two intronic markers, rs12255372 and rs7903146, are associated with the disease across multiple, ethnically diverse populations [87,88,89,90,91,92,93,94,95,96,97,98,99,100]. Because *TCF7L2* is expressed in pancreatic β-cells, and insulin secretion is reduced in individuals with the risk alleles at rs12255372 and rs7903146, carriers of these alleles may respond sub-optimally to sulfonylurea therapy due to decreased β-cell function [101]. A study involving 4469 participants from the Genetics of Diabetes Audit and Research Tayside (GoDARTs) provided evidence in support of this hypothesis by finding that individuals with the variant TT genotype at rs12255372 were less likely to respond to sulfonylurea treatment with a target HbA1c < 7% compared to carriers of the GG genotype (57% *vs.* 40%) [101]. Further, individuals with the TT genotype were much less likely to achieve a target HbA1c of 7% within one year of initiating sulfonylurea treatment compared with carriers of the GG genotype [101]. Similar results were observed with marker rs7903146. These results suggest that the *TCF7L2* locus may not only affect susceptibility to T2D, but may also modulate response to sulfonylurea therapy; in both cases, the pathophysiology likely stems from impaired insulin secretion due to deteriorating β-cell function.

The third gene, nitric oxide synthase 1 adaptor protein (*NOS1AP*), was examined as a candidate for sulfonylurea response due to previous findings of association between the rs10494366 marker and QT interval duration [102]. Association between this variant and T2D in patients being treated with calcium channel blockers has also been reported [103]. In the investigation of rs10494366 and sulfonylurea response, prescribed doses of glibenclamide were higher in individuals carrying the TG genotype compared with those with the TT genotype [104]. Provocatively, the G allele in glibenclamide users was associated with increased mortality risk compared to those with the TT genotype, while the same allele in tolbutamide and glimepride users was associated with a decreased mortality risk [104]. Replication of these findings should yield greater insight into the mechanism by which rs10494366 genotype differentially affects mortality risk in a sulfonylurea-agent manner.

### 2.2. Biguanides (Metformin)

#### 2.2.1. Background

Metformin ameliorates hyperglycemia by decreasing hepatic glucose output and gastrointestinal glucose absorption and improving insulin sensitivity [105], and is often the first drug used to treat newly diagnosed T2D in the United States [106]. However, glycemic response to metformin is variable, and approximately 35-40% of patients receiving the drug do not achieve acceptable control of fasting glucose levels [107,108]. Metformin is not metabolized, but instead undergoes rapid renal elimination via filtration in the glomerulus and net secretion in the proximal tubules [109]. The genetic component contributing to variation in renal clearance of metformin is >0.9, suggesting that genetic factors underlie variability in elimination of this drug [110,111]. 

#### 2.2.2. Mechanism of action

The molecular mechanisms underlying metformin action are initiated by the drug’s activation of adenosine monophosphate (AMP)-activated protein kinase (AMPK), which leads to suppression of glucose production via gluconeogenesis and increased peripheral glucose uptake [112,113]. Inhibition of hepatic gluconeogenesis by metformin occurs through AMPK-dependent regulation of the orphan nuclear receptor small heterodimer partner, SHP [114], and a protein-threonine kinase (LKB1), which phosphorylates and activates AMPK, is critical for the glucose-lowering effects of metformin in the liver [115]. Metformin is also known to exert metabolic effects through AMPK-independent mechanisms, but to date, these actions have only been observed in heart tissue [116]. Currently, the mechanism by which metformin increases AMPK activity, and hence, exerts its therapeutic effects, remain only partially understood. 

In addition to these effects, metformin may also exert a direct effect on pancreatic β-cells. In humans, metformin causes increased insulin release in response to glucose [117] and may help to preserve β-cell function [16]. *In vitro* studies using rat islets found that metformin restored insulin secretion to β-cells that had been impaired by chronic exposure to high levels of glucose and free fatty acids [118]. The molecular mechanisms by which metformin may exert a beneficial effect on β-cell function, however, remain unknown.

#### 2.2.3. Organic cation transporters and related proteins

Metformin is a hydrophilic organic cation (pK_a_ 12.4) that serves as a substrate for organic cation transporters (OCTs), including OCT1, which is expressed in the liver, and OCT2, which is expressed most abundantly in the kidney [119,120,121,122]. Correspondingly, OCT1 and OCT2 transport metformin into hepatocytes and renal epithelium, respectively, while the multidrug and toxin extrusion 1 protein (MATE1) facilitates metformin excretion from these cells into bile and urine, respectively. Drug transporter gene polymorphisms may underlie variation in drug response [110], and a number of studies have focused on the genes encoding the OCTs as mediators of variability in glycemic response or renal elimination of metformin. OCT1 and OCT2 belong to the SLC22A family of solute carriers and are encoded by the *SLC22A1* and *SLC22A2* genes, respectively. MATE1 is encoded by the *SLC47A1* gene.

Animal studies have firmly delineated biological relationships between metformin and its transporters. For example, in OCT1^-/-^ mice, significantly less metformin is distributed to the liver and small intestine compared to control mice [121]. However, there were no significant differences in kidney distribution and urinary excretion between null and control animals, which is consistent with OCT2, not OCT1, being the protein primarily responsible for renal elimination of metformin [121]. Additional *in vivo* studies showed no differences in plasma levels or pharmacokinetics of metformin between OCT1^-/-^ and control mice, but null animals showed significantly lower amounts of metformin accumulation in the liver and decreased hepatic AMPK activation [123]. Metformin also reduced fasting glucose levels in control, but not OCT1^-/-^ mice, following administration of a high-fat diet [123]. Metformin concentration in the blood tended to be higher in OCT1^-/-^ mice, although 24-hour plasma concentration-time profiles were similar between knockout and control animals [124]. In hepatocytes isolated from OCT1^-/-^ mice, metformin effects on AMPK activation and gluconeogenesis were reduced compared to cells cultured from OCT1^+/+^ mice [123]. 

##### 2.2.3.1. *SLC22A1*

Uptake of metformin into hepatocytes by OCT1 is a critical step for achieving its hypoglycemic effects; thus, variants in *SLC22A1* may be expected to contribute to differential glycemic response to the drug. Shu *et al.* [123] were the first to address this possibility by investigating 4 non-synonymous *SLC22A1* variants (*i.e.* R61C, G410S, 420del, and G465R: all of which are associated with reduced OCT1 function) in 21 healthy volunteers given metformin. In these individuals, no association between *SLC22A1* genotype and plasma glucose levels or AUC after OGTT was observed; however, following metformin dosing, volunteers carrying *SLC22A1* risk alleles had significantly higher plasma glucose levels and greater AUC for most of the sampling time points compared to those carrying wild type alleles. Insulin levels in individuals carrying risk alleles were also higher 2 hours after glucose administration compared to those with wild type alleles. *In vitro* characterization revealed that these variant alleles, particularly the 420del allele, had reduced activity for metformin. In a second study, Shu *et al.* [124] examined the same individuals with known *SLC22A1* genotypes (R61C, G401S, 420del, and G465R) and found that plasma metformin concentration tended to be higher in individuals carrying *SLC22A1* risk alleles *versus* wild type allele carriers.; these individuals also had a significantly higher maximal plasma concentration of metformin. 

The role of *SLC22A1* genotypes in response to metformin was also assessed in patients stratified according to drug response following 3 months of treatment [125]. In this study, responders to metformin were defined by a reduction in HbA1c levels > 0.5% from baseline and non-responders were those for whom meformin therapy had been discontinued within 3 months and/or after another hypoglycemic drug had been added to the therapy. Of the 11 *SLC22A1* markers genotyped, none showed differences in allele frequency between the two groups [125]. These findings differed from those reported earlier [123], but there were underlying differences in study design, ethnicities of samples, and measured outcome between the two studies, making comparisons difficult. In addition, there were no genotyped markers in common between the two studies. Finally, sample sizes in both studies were quite small, and as such, likely underpowered to detect association. In light of these discrepancies, evaluation of findings from these investigations in a population study involving large numbers of patients would be worthwhile. 

In addition to investigation of effects of *SLC22A1* variants on glycemic response to metformin, other studies have investigated genetic association between these markers and variation in renal clearance of the drug [126]. In a sample of 103 healthy male Caucasians given a single 500 mg dose of metformin, 2 *SLC22A1* markers, rs1867351 (promoter) and rs45476695 (Gly465Arg), were associated with renal clearance of the drug. Interestingly, carriers of zero, one, or two reduced-function alleles (*i.e.* R61C, 420del, S401G, and G465R) had higher mean renal clearances of metformin, which is inconsistent with previous findings suggesting a lower clearance rate for the drug [123]. While these studies are not directly comparable as metformin dosage and study designs were different between the two, the study by Tzvetkov *et al.* [126] had a significantly larger sample size, including 4 homozygous carriers of the low-activity alleles, which improves the power to detect an association. 

OCT2 is expressed in the basolateral membrane of the renal epithelium and transportation of metformin over this membrane may be the first step to tubular secretion. Renal OCT2 may be critical for regulating the accumulation of metformin in the kidney in rats and may play a more substantial role in the pharmacokinetic profile of the drug than OCT1 [127]. The variant T allele at marker 808G > T in *SLC22A2*, was associated with reduced renal clearance of metformin and lower renal tubular clearance in the presence of the OCT2 inhibitor cimetidine in 15 healthy Chinese individuals [128]. A subsequent study conducted by Song *et al.* [129] in 26 healthy Korean individuals found that three *SLCA22A2* variants (596C > T, 602C > T, and 808G > T) were associated with decreased renal excretion and increased plasma concentration of metformin, although the 596C > T and 602C > T markers have a very low minor allele frequency and appear to be specific to individuals of Asian ancestry [130,131]. Similarly, in a study of 23 healthy individuals of Caucasian and African American ethnicities, individuals with the G/T genotype at the *SLC22A2* 808G > T locus had reduced rates of both renal clearance and net secretion compared to carriers of the wild type G/G genotype [132]. In contrast to these three studies, an investigation conducted by Tzvetkov *et al.* [126] in 103 healthy participants, did not report statistically significant evidence for association between 14 *SLC22A2* markers, including 808G > T, and renal metformin clearance. It is possible that *SLC22A2* markers are largely important for the renal elimination of metformin in individuals of Asian ancestry, which may explain the discrepancies between the first two reports and this study; however, the latter had a substantially larger sample size compared to the other studies, which likely accounts for the majority of the differences among the reported findings. Despite these differences, findings reported for *SLC22A2* polymorphisms may have clinical relevance, but larger prospective studies in individuals with T2D are critical before the impact of these markers on clinical pharmacokinetics and therapeutic effects can be established.

##### 2.2.3.2. *SLC47A1*

The *SLC47A1* gene encodes the MATE1 protein. MATE1 is located in the bile canicular membrane in the hepatocyte and the brush border of the renal epithelium, where it functions to excrete metformin through the bile and urine [133]. MATE1 is colocalized with OCT1 and OCT2 in the hepatocyte and renal epithelium, respectively [133]. Because of its role in metformin excretion, the function and activity of MATE1 may contribute to the variability in response to the drug, yet little is known of the effect of genetic variants in *SLC47A1* and metformin response. To date, only one study has investigated this gene. In that study, 12 tagging SNPs in the *SLC47A1* gene were genotyped in 116 patients using metformin, and association was observed with only one marker, rs2289669, and metformin response, as defined by a decrease in HbA1c levels [134]. For each minor A allele in this study, the decrease in HbA1c level was 0.3%. In contrast, however, a study by Tzvetkov *et al.* [126] did not observe association between *SLC47A1* variants and renal clearance of metformin. At this time, the clinical impact of both rs2289669 and *SLC47A1* needs to be evaluated further and confirmed in other populations.

### 2.3. Thiazolidinediones

Thiazolidinediones (TZD) are a class of insulin-sensitizing drugs that are agonists for the nuclear receptor peroxisome proliferator-activated receptor-γ (*PPARG*). The first TZD, troglitazone (Rezulin©), was approved for use in the United States in 1997, immediately followed by pioglitazone (Actos©) and rosiglitazone (Avandia©). The exact mechanism by which TZDs act has not been clearly delineated; however, data indicate that TZDs increase insulin sensitivity by both direct and indirect effects on adipose tissue and muscle [135]. Troglitazone was removed from the market in 2000 due to hepatotoxicity [136], but pioglitazone and rosiglitazone remain on the market.

#### 2.3.1. Mechanism of Action

There are three known forms of the nuclear receptor PPAR; PPAR-α, PPAR-γ, and PPAR-δ, which are encoded by distinct genes and have disparate tissue expression patterns [137]. TZDs are selective agonists for *PPARG2*, which is predominantly expressed in adipose tissue, and appear to have minimal activity on *PPARG1* or *PPARG3* [138]. TZD stimulation of PPARG2 results in increased adipocyte differentiation [138] and has been shown to reduce hyperglycemia in patients with T2D [139,140,141]. There is also evidence for other effects of TZDs not mediated through adipose tissue [142,143,144,145].

#### 2.3.2. TZD Kinetics

Each TZD has specific kinetic characteristics. For example, the binding affinity of troglitazone to PPARG is approximately 2-fold higher than that for pioglitazone, and 20-fold higher than rosiglitazone. The binding affinities appear to track with the efficacy of each compound to lower glucose levels [146,147]. The differences in binding affinity result in a half-life for troglitazone that is 2 to 4 times longer than that for pioglitazone [135,138,146]. TZDs appear to be metabolized through the family of cytochrome P450 enzymes. Troglitazone is metabolized into sulphate and glucuronide conjugates and a quinine-type metabolite [148,149], and its metabolism appears to inhibit activi­ties of other cytochrome P450 enzymes, suggesting it may interact with other medications. In contrast, pioglitazone is metabolized into 5 metabolites, mainly by CYP3A4, CYP2C8, and CYP2C9, and three of these metabolites appear to be active [150]. Unlike troglitazone, pioglitazone does not appear to inhibit activity of other cytochrome P450 enzymes and therefore is expected to have few drug interactions [151,152].

#### 2.3.3. TZD Efficacy and Risks

Numerous clinical trials have demonstrated the efficacy of TZDs to reduce hyperglycemia and HbA1c levels, and improve insulin sensitivity [31,139,140,153,154,155]. Despite troglitazone being withdrawn from the market, both pioglitazone and rosiglitazone remain treatment options for patients with T2D. Early in clinical trials TZD monotherapy was found to be associated with both weight gain [156,157,158,159,160] and edema [157,158,161], and these side effects raised concerns regarding secondary cardiovascular effects of TZD monotherapy. Currently, TZD monotherapy is not recommended in patients at-risk for congestive heart failure. 

Data regarding the cardiovascular risk profile of TZDs remains controversial. In 2007, a meta-analysis of available data from clinical trials of rosiglitazone appeared to confirm increased risk of myocardial infarction [162]. The study also reported an increased risk of death from cardiovascular causes that was “borderline” significant. A few months later, this report was followed by a second meta-analysis, which concluded that in contrast to rosiglitazone, pioglitazone was associated with a *lower risk* of death, myocardial infarction, and stroke [163]. The study also concluded that while pioglitazone therapy was associated with an increased risk of heart failure, this association was not accompanied by an increased risk in mortality. The controversy regarding the cardiovascular risk profile of TZDs continues and clearly requires carefully designed studies to directly assess this question [164,165,166].

Most recently, a trial examining the glycemic durability of different T2D pharmacotherapies, noted an increased risk of bone fractures in women participating in the rosiglitazone arm of the trial [167]. This increased risk was confirmed in a more comprehensive follow-up study [168] and raises new concerns regarding the risk profile for TZDs. 

#### 2.3.4. Prevention

The efficacy of TZD monotherapy to treat T2D led to the question of whether treating at-risk individuals could be an effective intervention to reduce T2D risk. The TRoglitazone In the Prevention Of Diabetes (TRIPOD) was the first trial to successfully demonstrate that TZD monotherapy in at-risk individuals could significantly reduce risk for future T2D [169]. Buchanan and colleagues demonstrated a significant reduction in risk for future T2D in Hispanic women with a previous history of gestational diabetes mellitus, a group shown to have an extremely high risk of T2D [170]. Buchanan and colleagues [5] recapitulated the observations in TRIPOD for pioglitazone (PIoglitazone In the Prevention Of Diabetes; PIPOD). These observations have since been replicated in larger clinical trials such as the Diabetes Reduction Assessment with Ramipril and Rosiglitazone Medication (DREAM) and the Diabetes Prevention Program [2,4].

#### 2.3.5. Response

The mechanism of action, kinetics, and risk profile of TZDs create a vast list of possible genetic targets that may contribute to differences in both drug response and risk profiles. Clinical trials testing the efficacy of TZD monotherapy have reported varying response rates [141,171,172,173]. The variability in response may, in part be due to the varying definitions of “response,” which ranged from changes in fasting glycemia or HbA1C to changes in measures of insulin sensitivity directly assessed using the glucose clamp or IVGTT. Few studies have made direct assessments of insulin sensitivity, but in those that did [1,169,174], lack of response to TZDs ranged from 30-40%. These observations suggest a substantial fraction of treated individuals will not respond to the insulin-sensitizing effects of TZDs. Theoretically, clinical or physiologic characteristics could predict which individuals will or will not respond to TZD therapy. For example, very obese individuals or those at the extremes of the distribution of insulin resistance, are the most likely candidates to benefit the most from TZD therapy. However, to date, pre-treatment clinical or physiological characteristics have not been useful in distinguishing responders from non-responders to TZD therapy [4,5,169]. 

While data on general response rates to TZDs are available, to our knowledge there are no studies specifically examining differences in response among the different TZDs within a single individual. Although the family of TZDs has common structural components, they are structurally different chemical compounds [175]. Physicians treating patients with TZDs provide anecdotal evidence regarding differential TZD response within single patients, but there has not been a comprehensive study of within-patient response rates. Disparities in response among members of the same family of drugs could hold important clues to the role of genetic variation in the response mechanism. The TRIPOD [169,176] and PIPOD [5] studies represent the only data we are aware of in which individuals were treated with both troglitazone and pioglitazone and response was assessed by examining changes in insulin sensitivity. These studies were not designed to specifically address differential response to different TZDs, thus there are a number of experimental design issues that confound the observations in this regard. However, when examining the small number of women who were treated with both compounds (n = 32), there was no concordance in terms of response rates (Watanabe RM, unpublished data), suggesting that different clusters of genetic variation may underlie differential response to these two TZDs. 

Non-response rates in TZD therapy appear to be similar across diverse populations suggesting little to no contribution from environmental exposures to differences in response. Furthermore, issues related to ethnic/racial differences or compliance is not likely to significantly contribute to response given the observed similarity across very diverse studies. These observations led to the hypothesis that genetic variation may be an important and significant contributor to the TZD response mechanism. However, given the observation that individuals may have differential response to different TZDs, it is possible that gene variants that underlie response to one TZD may not contribute to response to another.

##### 2.3.5.1. *PPARG*

An obvious genetic target to assess pharmacogenetics of TZD therapy is PPARG, the target of TZDs. A specific common variant in *PPARG* (rs1801282; Pro12Ala) was initially shown to be associated with T2D and insulin sensitivity by Deeb *et al*. [177], and subsequently confirmed in a large meta-analysis [178]. This variant was a logical target as potentially having a role in response to TZD therapy. Snitker *et al.* [179] were the first to test this hypothesis in the TRIPOD study and showed that rs1801282 was not associated with a troglitazone-induced improvement in insulin sensitivity as assessed by the intravenous glucose tolerance test and minimal model analysis. Similar lack of association between this variant and response to troglitazone therapy assessed as a change in HOMA-IR [180], an indirect measure of insulin sensitivity, was reported by the Diabetes Prevention Program (DPP) [181]. These observations extend to pioglitazone therapy where no association was observed between rs1801282 and improvement in fasting glycemia or HbA1c [182].

The lack of association with this single T2D diabetes susceptibility variant did not exclude the possibility that variation elsewhere in *PPARG* could be contributing to TZD response. Wolford *et al.* [183] sequenced the coding region of *PPARG* and tested variants for association with TZD response in the TRIPOD study. Among the 133 SNPs they identified, eight showed evidence for association with response to troglitazone monotherapy, which was defined as an improvement in insulin sensitivity measured using the intravenous glucose tolerance test with minimal model analysis. The odds ratios for these associations ranged from 2.04 to 2.36. These observed odds ratios for troglitazone response are in stark contrast to the relatively small odds ratios observed for disease susceptibility (*cf.* [184]), but are consistent with other pharmacogenetic studies in which relatively large effect sizes are observed (*cf.* [185]). Another important observation was that SNPs showing association with TZD response as defined by a change in insulin sensitivity did not show evidence for association with change in fasting glucose. Therefore, had a change in glycemia been used to define response, no associations would have been observed. Because the glucoregulatory system is designed to tightly regulate glycemia, this metric may only be sensitive enough to detect large relatively large changes in TZD response. This is consistent with the observation that in the progression to T2D, large changes in glycemia are only observed when β‑cell failure ensues [186,187]. 

The associations observed in TRIPOD were not replicated in the DPP [181], which raises important considerations in both the conduct of and the comparison between pharmacogenetics studies. One might consider differences between the two studies, such as duration of treatment (3 months for TRIPOD and 1 year for the DPP), mean age (36 yrs for TRIPOD *vs.* 51 yrs for the DPP), ethnic/racial composition of the study participants (Hispanic for TRIPOD *vs.* a mix for the DPP), and T2D risk (previous gestational diabetes mellitus for TRIPOD *vs.* impaired glucose tolerance for the DPP), as potential explanations for the divergent association results. However, they do not negate the fact that both studies showed a significant effect of troglitazone to reduce risk for T2D and both studies observed responders and non-responders. One potential explanation is statistical power. The sample size for TRIPOD (total n = 93) was substantially lower than that for the DPP (total n = 3548), suggesting that the observations from the TRIPOD study represent false positives. However, a priori power calculations suggest TRIPOD had sufficient power to detect effects within the range of the observed associations. A second explanation is the difference in how response was quantified in the two studies. Since troglitazone is an insulin-sensitizing agent, both studies assessed TZD response as a change in insulin sensitivity. TRIPOD employed a direct measure, while the DPP used an indirect measure of insulin sensitivity. Although direct and indirect measures of insulin sensitivity typically show very strong correlation, when specifically examining environmental *vs.* genetic correlations, the genetic correlation between HOMA-IR and minimal model-based insulin sensitivity is much weaker, suggesting they do not capture the same genetic information [188]. This is consistent with simulation studies showing that indirect measures of insulin sensitivity are confounded by insulin secretion and may not appropriately reflect changes in insulin sensitivity [189]. 

##### 2.3.5.2. *Adipokines*

In rodents TZD therapy results in a PPARG-mediated increase in new adipocytes of small size [190], potentially explaining the modest increase in body weight that paradoxically accompanies the reduction in hyperglycemia in humans [140,141,171,172,174,191]. It has been hypothesized the smaller-sized adipocytes, which are deposited within the subcutaneous depot, are more sensitive to insulin, and therefore able to efficiently take up glucose [192] and triglycerides, and have lower rates of lipolysis. TZDs also significantly reduce triglyceride content in adipose tissue, skeletal muscle, and liver, and increase leptin concentrations [193,194]. Together, these changes lead to a decrease in circulating free fatty acids (FFA), which reduces FFA-induced insulin resistance in skeletal muscle. It has also been shown that TZD therapy alters concentrations of other adipokines, such as leptin, adiponectin, and TNF-α [135,151,190,194,195]. Data also suggests that troglitazone-induced changes in insulin sensitivity are not associated with changes in total adiponectin concentration, but with changes in the high molecular weight sub-fraction [196]. Responders to troglitazone showed a significant increase in the high molecular weight sub-fraction, while non-responders showed no change [196]. These observations make adiponectin (*ADIPOQ*) an attractive target for genetic analysis.

**Table 1:** Selected association results between rs2241766 in *ADIPOQ* and response to rosiglitazone therapy extracted from Table 4 from [77].

	T/T	T/G	G/G	p-value*
**Total sample size**	86	55	25	0.032
**Change in Fasting Glucose (mM)**	1.63±2.19	1.79±2.74	0.25±2.95	
**Change in HbA1c (%)**	0.83±1.13	0.87±0.92	0.05±1.43	0.006
**Change in Adiponectin (μg/mL)**	5.52±5.03	3.68±4.98	1.67±4.45	0.002

* p-value for the test of differences among genotypes.

Kang *et al*. [197] examined the association between variation in *ADIPOQ* and response to rosiglitazone assessed by changes in fasting glucose and HbA1c in Korean patients with T2D. Two variants in *ADIPOQ*, rs1501288 and rs2241766, were associated with reduced changes in both fasting glucose and HbA1c in response to 12 weeks of rosiglitazone therapy. A summary of selected results for rs2241766 extracted from this study are presented in Table 1. It is noteworthy that the statistical analysis in this study was based on examination of differences among genotypes by *ANOVA* and by individual comparison of the G/T and T/T means to the G/G homozygotes, rather than assuming a specific underlying genetic model. Individuals homozygous for the G allele had the smallest change in fasting glucose, HbA1c, and adiponectin suggesting these individuals would not gain benefit from rosiglitazone therapy. Similarly, individuals homozygous for the rs1501288 G allele also had the smallest changes in these metrics and may not benefit from rosiglitazone therapy. It is interesting to note that although HOMA-IR was assessed in this study, change in HOMA-IR was not formally tested for association with *ADIPOQ*. Baseline HOMA-IR showed no differences among genotypes for both SNPs examined, but it is unclear whether change in HOMA-IR would have supported the results observed with change in fasting glucose, HbA1c, and adiponectin.

Additional information regarding the underlying genetic model driving these results can be extracted from the results of this study. As can be seen in Table 1, the association between rs2241766 and change in adiponectin concentration appears to follow an additive genetic model; the change in concentration becoming smaller with each copy of the G allele. However, both change in fasting glucose and HbA1c do not show evidence for additivity and may be more consistent with a dominant (T allele) or recessive (G allele) genetic model. Unfortunately, specific genetic models were not tested in this study. 

The association between *ADIPOQ* and response to rosiglitazone therapy was also examined in a small case-control (225 cases, 120 controls) sample of Chinese subjects [198]. In contrast to the findings by Kang *et al.* this study found no evidence for association between rs2241766 and change in fasting glucose or HbA1c. They did observe an association with change in adiponectin concentrations, but in contrast to the study of Kang *et al.* changes in adiponectin concentration were larger in individuals carrying the G allele. Interestingly, rs2241766 did show a marginal association with change in fasting insulin in these Chinese subjects, with the percentage change being smaller in individuals carrying the G allele. If one took fasting insulin as an indicator of insulin resistance, this would be consistent with these individuals potentially being non-responders to rosiglitazone therapy.

Additional studies have shown varying levels of evidence for association between response to TZDs and leptin [199], TNF-α [199], and resistin [200]. Although these results are relatively under-powered, they point to the adipokine signaling system and a potential neuro-regulatory mechanism underlying TZD response. Independent replication of these findings in larger sample sizes will be required before they can be accepted as valid associations.

##### 2.3.5.3. *Cytochrome P450 Enzymes*

As noted above, previous to the era of genome-wide association, pharmacogenetics tended to focus on drug pharmacokinetics, due to the relative lack of pharmacodynamic information as phenotypes. The metabolism and kinetics of TZD compounds have been studied extensively, and several groups have examined the association between variation in genes encoding cytochrome P450 enzymes and TZD response. 

Kirchheiner and colleagues [201] were among the first to examine association between pharmacokinetic characteristics of rosiglitazone and variation in *CYP2C8*. They performed pharmacokinetic studies of rosiglitazone in 31 subjects and tested whether these characteristics were associated with *CYP2C8* genotype. The clearance rate of rosiglitazone was greater in CYP2C8*1/*3 and *3/*3 individuals compared to CYP2C8*1/*1 individuals. Although a specific genetic model was not tested in this study, the clearance rate appeared to increase with each copy of the *3 allele. This result was consistent whether rosiglitazone was given as a single 8 mg dose or over a two week treatment period (8 mg/day). However, there was no association between *CYP2C8* genotype and glucose levels, suggesting that variation in *CYP2C8¸* while affecting rosiglitazone pharmacokinetics, did not modify the drug’s insulin sensitizing effects. Similar to the observations by Kirchheiner and colleagues, Tornio *et al.* [202] showed that plasma pioglitazone concentrations, assessed as area under the curve, were higher in CYP2C8*1/*3 and *3/*3 compared to *1/*1 individuals. Although drug clearance rates were not estimated in this study, the results are consistent with differences in drug clearance and with the observations for rosiglitazone made by Kirchheiner *et al.* Tornio and colleagues did not assess whether *CYP2C8* genotype had any effects on glucose levels or other glucoregulatory measures. 

The association between pharmacokinetics of rosiglitazone and *CYP2C8* was replicated in a second study by Aquilante *et al.* [203]. However, in addition to *CYP2C8*, they also examined the association between variation in *SLCO1B1* (solute carrier organic anion transporter family, member 1B1) and rosiglitazone pharmacokinetics. SLCO1B1 is a liver-specific member of the organic anion transporter family that mediates sodium-independent uptake of a variety of endogenous compounds and is involved in the removal of various drugs. Variation in *SLCO1B1* has been shown to be associated with slow response to pravastain [204] and with statin-induced myopathy [205]. *SLCO1B1* variants were not statistically associated with rosiglitazone pharmacokinetics the combined *1A/*1B and *1B/*1B group tended to have a lower rosiglitazone clearance rate compared to the *1A/*1A genotype group (33 ± 9.2 *vs.* 40.9 mL/h/kg; p = 0.35), which resulted in the combined group having slightly higher plasma rosiglitazone concentrations.

## 3. Future Approaches for Pharmacogenetics Studies of Anti-Diabetes Drugs

Pharmacogenetic studies of T2D therapies lags significantly behind other complex diseases, despite the fact that pharmacologic interventions for T2D have been studied extensively at both the clinical and epidemiologic levels. For example, the sulphonylurea class of drugs has been in clinical use since the 1950s with the so-called “second generation” sulphonylureas approved for use in the US in 1984. Despite this long history, a simple PubMed search using the terms “pharmacogenetics” and “diabetes” yields only 88 original scientific papers spanning a 43 year period (1967-2010). If one replaces “diabetes” with “cardiovascular disease”, the same search yields 330 original scientific papers covering a 42 year period (1968-2010). Sadly, a search of review articles for diabetes pharmacogenetics yields 81 reviews, suggesting that while the basic idea of pharmacogenetics has percolated in scientific minds for decades, there has been little translation into the clinical research setting.

Rapid advances in genomic technologies have revolutionized studies of human genetics. As of this writing, 38 loci underlying susceptibility to T2D have been identified, mostly in populations of northern European ancestry [206,207,208,209,210,211,212,213,214,215,216,217]. In contrast to the very small effect sizes of diabetes susceptibility loci, effect sizes for response to medications or adverse events may be substantially larger (2- to 50-fold), thus making it feasible to perform genome-wide association studies for such phenotypes without the need to obtain extremely large sample sizes. This point was illustrated by Nelson and colleagues who demonstrated that statistically significant, genome-wide associations can be detected with sample sizes in the low hundreds for certain adverse events [185]. Thus, it should be possible to take existing drug trial data where DNA is available and perform genome-wide association analyses, much in the manner that was done for T2D. However, given the relatively smaller number of drug trials performed using identical patient ascertainment and treatment protocols, replication of primary findings from pharmacogenetics-based GWASs may be problematic. Replication of primary GWAS signals should pay careful attention to heterogeneity analysis or consider using a random effects meta-analysis rather than a fixed effects approach that has been traditionally implemented for disease-based GWAS. Regardless, DNA should be routinely collected in future drug trials to facilitate pharmacogenomic studies.

That said, the era of genome-wide association may be short-lived given the advent of “next generation” sequencing [218]. Although this technology, and its variations like RNA-sequencing, is currently expensive, the cost-per-sample is rapidly dropping and soon may be the standard technology for assessing human genetic variation. Such technologies can provide rapid interrogation of >99% of the human genome covering both common and rare variants that may contribute to drug response or adverse events. Indeed, like complex diseases, it is likely that rare variants of high penetrance may contribute to drug response and sequencing is currently the only technology that allows efficient identification of such variation. Obviously, such technology would be overkill in the clinical setting, but is an obvious research tool to identify genetic variants that may be predictive of drug response. If predictive variants can be identified, it should be possible to develop a specific SNP chip that would be more appropriate for the clinical setting. In addition, one should consider the potential contributions of other genetic variation, such as copy number variants, insertion/deletions, and epigenetic modification, to drug response.

Regardless of what technologies are applied to pharmacogenetic studies, a critical feature of any pharmacogenetic study of T2D is the phenotype. As noted above, a variety of endpoints have been used to define response to T2D therapies. The most common has been changes in fasting glucose and/or HbA1C. This is a logical choice from a clinical perspective, given that the ultimate goal for a clinician is normalization of glycemia. However, this may not be the optimal choice to understand the role of genetic variation on drug response. First, as noted above, each class of T2D therapies operates through different mechanisms of action, including insulin secretion, hepatic glucose output, insulin sensitivity, *etc.* Therefore, any change in fasting glucose or HbA1c is secondary to the drug effect at the molecular target and changes in these metrics may not accurately reflect the actions of the drug, given that multiple mechanisms are simultaneously working to regulate glucose levels. This effect is best illustrated in the examination of changes in fasting or 2-hour glucose levels as individuals progress towards T2D. Buchanan and colleagues were among the first to show that both fasting and 2-hour glucose rise minimally in individuals who did not develop T2D over a 5-year follow-up period; 1 mg/dl per year and 4 mg/dl per year, respectively [187]. Although changes in these metrics were larger in individuals who eventually developed T2D over the same 5-year follow-up (19 mg/dl per year and 28 mg/dl per year, respectively), in both groups glucose levels were maintained in a relatively narrow range, mostly due to compensatory insulin secretion. However, as β‑cell compensation approached and then fell below 10% of normal, there was a rapid rise in both fasting and 2-hour glucose in those individuals who developed T2D. The same pattern of changes in glucose was observed in Pima Indians [186]. Therefore, accurate assessment of the specific physiologic parameter targeted by the given T2D therapy may be the optimal measure of drug response compared to fasting glucose or HbA1c.

Finally, genetic associations only provide information regarding specific genetic markers that may be predictive of drug efficacy. To date, association studies have not formally assessed specificity or sensitivity. While these metrics are not fully informative in genetic association studies, they do provide some information on the utility of a given marker as a predictive tool. Furthermore, there has not been a study to jointly examine all variants for a given therapy to assess whether the joint information accounts for a greater proportion of the variability in drug response compared to the individual markers alone. Clearly, prospective studies testing the power of genetic markers to predict drug response are requisite to fully endorse their introduction into the clinical care setting.

## 4. Conclusions

Pharmacogenetics research provides a means to better understand and improve on pharmacotherapy. However, pharmacogenetic studies of T2D therapies lag behind those for other complex diseases, despite the fact that pharmacologic interventions for T2D have been studied extensively at both the clinical and epidemiologic levels. Among the studies that have been conducted, several have identified variants that are potentially associated with differential response to anti-diabetes medications; these preliminary results are promising and warrant investigations in larger, well-designed cohorts to assess their potential roles in optimal drug selection and individualized pharmacotherapy in patients with T2D. At this time, larger, well-powered studies with clearly defined outcomes and utilizing a global approach are needed, as they will not only be more informative than extant candidate gene investigations, but will also be necessary to define the array of genetic variants that may underlie drug response. Such results will likely enable achievement of optimal glucose control, improvement of therapeutic efficacy, and reduction in risk of adverse drug events in at-risk patients, which together will lead to personalized treatment strategies for all individuals with T2D. 
